# Laparoscopic-assisted percutaneous herniorrhaphy as an alternative to open surgery technique in farm swines

**DOI:** 10.1371/journal.pone.0256890

**Published:** 2021-09-03

**Authors:** Przemysław Prządka, Bartłomiej Liszka, Agnieszka Antończyk, Piotr Skrzypczak, Zdzisław Kiełbowicz, Dariusz Patkowski

**Affiliations:** 1 Department and Clinic of Surgery, Faculty of Veterinary Medicine, Wroclaw University of Environmental and Life Sciences, Wroclaw, Poland; 2 Department of Paediatric Surgery and Urology, Medical University of Wroclaw, Wroclaw, Poland; Ohio State University Wexner Medical Center Department of Surgery, UNITED STATES

## Abstract

**Background:**

Despite numerous experimental studies presenting laparoscopic treatment of inguinal hernia in a pig model so far no described technique has been used in clinical patients of this species. Minimal invasiveness and the simplicity of closure of the inguinal canal using the Percutaneous Internal Ring Suturing (PIRS) technique makes it the world’s first technique for laparoscopic treatment of inguinal hernia in pigs as clinical patients.

**Aim:**

This study aims to assess the applicability and effectiveness of the laparoscopic PIRS technique in the treatment of inguinal hernia in pigs as clinical patients and to compare the PIRS technique with the open surgery technique, which is currently being used.

**Methods:**

The study was conducted on 22 non-castrated male pigs with inguinal hernia (clinical patients), divided into two equal groups: PIRS and open surgery (OS). In the PIRS group, the inner inguinal ring was closed with an optical trocar inserted at the umbilicus level and an injection needle with a suture material inserted percutaneously over the inguinal canal. The suture material was threaded through the inner inguinal ring and then tied, leaving the knot under the skin. As a result to this the inguinal canal was closed. In the OS group the procedure was performed with open access above the inguinal canal where, after dissection of the vaginal processus and reducing the contents of the hernia to the abdominal cavity, it was ligated as close to the inguinal canal as possible, and the wound was then closed in layers.

**Results:**

All operated pigs returned to full fitness immediately after recovery from anesthesia. There was one case of hernia recurrence in the PIRS group. In the OS group all the operated pigs had a temporary swelling of the postoperative wound and the scrotum on the side of the operated inguinal hernia, which was not found in the PIRS group.

**Conclusions:**

The effectiveness of the PIRS technique is comparable to that of open surgery. Considering the simplicity of the PIRS procedure and its minimal invasiveness, this technique may be used as an alternative to the open technique in the treatment of inguinal hernias in pigs not subjected to surgical castration.

## Introduction

Surgical castration of piglets is routinely performed in most pork producing countries. One of its main goals is to eliminate the characteristic unpleasant smell of pork after the slaughter of male pigs [1]. Alternative solutions to avoid surgical castration are currently and increasingly being searched. This is due to, inter alia, complications related to surgical castration and the stress and suffering of pigs, that are related to the procedure. Also, there is increasing pressure from consumers who pay attention and demand that the meat they eat come from animals that are not exposed to unnecessary suffering [2]. An examples of measures to bypass surgical castration are: the slaughter of male individuals of lower body weight before reaching sexual maturity [3], immunization of male pigs against gonadotropin-releasing hormone (or so-called immunocastration) or sperm sexing to obtain only female individuals for rearing porkers [2].

The inguinal and scrotal hernia are variants of a defect that consists of pathological displacement of the abdominal organs through the inguinal canal [4]. The frequency of inguinal hernia, noted most often in males, varies depending on the source—from 0.7% up to even 15.7% of the entire pig population [4–6]. At the same time, the scrotal hernia is one of the most frequently reported congenital defect of the aforementioned species, causing economic losses in breeding. The herniated individuals cannot be castrated on the standard day of early castration, which causes further difficulties [7]. Untreated chronic inguinal/scrotal hernia causes a reduction in daily increments, peritonitis and (in extreme cases) leads to the entrapment and strangulation of the intestines, threatening the health and life of sick pigs [8–10].

The intensive search for an economically beneficial alternative to surgical castration and the pressure of consumers makes the traditional open surgery treatment of inguinal hernia during castration lose its importance. Therefore, according to the authors of this study, it seems necessary to introduce new, less invasive methods of surgical treatment of inguinal hernias in both breeding pigs and pigs kept as companion animals (pet pigs).

There are numerous publications in the literature presenting the possibilities of endoscopic surgery in the treatment of inguinal hernias using pigs as an experimental model for human medicine [11–13]. Unfortunately, due to economic reasons, none of the described techniques of laparoscopic hernia treatment has so far found clinical application, both in breeding pigs and in pet pigs.

Currently endoscopic surgery is more and more often used in the treatment of farm animals, an example of which is the use of a portable (compact) laparoscope for surgery in cows [14, 15] and horses [16].

An interesting and economically promising solution for the treatment of inguinal hernia using endoscopic surgery is the PIRS technique—Percutaneous Internal Ring Suturing, described in humans [17] and in dogs [18]. In the technique mentioned the inner inguinal ring is closed by a percutaneous suture material using an injection needle. The entire procedure is monitored using a laparoscopic camera inserted at the umbilicus [17–21]. The PIRS technique through the use of extra-corporeal suturing does not require the surgeon to have advanced laparoscopic skills, which makes it technically easy [18, 21–23].

According to the available knowledge of the authors, this work is the first publication in the world presenting the possibility of using endoscopic surgery in the treatment of inguinal hernia in pigs as clinical patients using the PIRS technique. The aim of this paper was to assess the application possibility of laparoscopic-assisted percutaneous herniorrhaphy procedure in treating inguinal hernias in farm pigs and to compare it to the currently used open surgery herniorraphy technique, while doing late castration. Highlighting the pros and cons of both methods in juxtaposition may allow us to tell if the PIRS technique could be the attractive alternative in pigs that are not being castrated.

## Materials and methods

The presented procedures were performed on patients of the Department and Clinic of Surgery at the Wrocław University of Environmental and Life Sciences after obtaining the written consent of the animal owners. The studies were approved by the II Local Ethical Committee for Experiments on Animals in Wroclaw (Resolution no. 011/2020/P2). All surgical procedures were performed on farm pigs by the same team of doctors.

The clinical trial was carried out on 22 non-castrated male Polish white fold pigs. All the pigs involved in the experiment were housed on pig farms throughout the study period and had a clinically proven scrotal hernia. The operated pigs were equally divided into two groups depending on the operating method used, i.e. open surgery (n = 11; OS group) and endoscopic surgery—Percutaneous Internal Ring Suturing (n = 11; PIRS group). Individual assignment to the group was determined by the owners’ consent to chosen procedure. Pigs operated with the PIRS technique were pharmacologically castrated (immunocastration, Improvac, Zoetis, Belgium), while classically operated pigs were surgically castrated during the surgical treatment of an inguinal hernia. The clinical evaluation of the described treatments effectiveness continued until the operated pigs reached their slaughter weight, that is for three months after the procedure. Additionally, the study assessed the time of anaesthesia, the duration of the procedure and the length of postoperative wounds. The time of anaesthesia was counted from the time of induction of general anaesthesia to the moment immediately after the end of the surgical procedure. The time of the procedure was counted from the first skin incision to the placement of the last skin suture, concluding the surgery. The length of postoperative wounds was measured using a tailor’s measure–Tables 1 and 2.

**Table 1:** Listing and additional information about operated pigs–open surgery group.

Group OPEN SURGERY							
Patient number	Weight (kilograms)	Side of the hernia	Anesthesia time (minutes)	Time of surgery (minutes)	The length of the wound above the inguinal canal (centimeters)	The length of the scrotum wound (centimeters)	Total length of the wound—inguinal canal + scrotum (centimeters)
1	32	right	26	19	6,5	4	10,5
2	35	left	22	16	6	3,5	9,5
3	20	right	30	21	5,5	3,5	8
4	35	left	32	28	7	3,5	10,5
5	35	left	33	29	7	4	11
6	27	left/right	42	35	6,5/6	0	12,5
7	18	left	25	22	5,5	3	8,5
8	12	right	30	25	4	3	7
9	13	right	20	15	4,5	3,5	8
10	20	left	19	12	5	3,5	8,5
11	40	left	27	22	7	4	11

**Table 2:** Listing and additional information about operated pigs–PIRS group.

Group PIRS						
Patient number	Weight (kilograms)	Side of the hernia	Anesthesia time (minutes)	Time of surgery (minutes)	Pneumoperitoneum time (minutes)	Length of the wound on the umbilicus (centimeters)
1	45	right	33	23	13	0,5
2	43	right	36	24	16	0,5
3	41	left	38	25	18	0,5
4	46	left	37	28	19	0,5
5	39	left	33	27	18	0,5
6	38	left	25	17	10	0,5
7	35	left	28	16	9	0,5
8	33	left/right	40	31	30	0,5
9	30	left	30	16	12	0,5
10	29	left	36	24	7	0,5
11	28	right	28	15	11	0,5

### Anesthesia, pre-and postoperative management

The animals were anesthetized intramuscularly with a mixture of ketamine (10mg / kg, Bioketan 100mg / ml, Vetquinol, Poland), medetomidine (0.03mg / kg, Cepetor 1mg / ml, CP-Pharma, Germany) and methadone (0.2mg / kg, Comfortan 10mg / ml, Eurovet Animal Health BV, The Netherlands). Then a catheter was inserted into the marginal vein of the ear. The animals were not intubated and breathed atmospheric air. Patients were monitored using the Mindray IMEC8 portable patient monitor (pulse oximetry, ECG, NIBP, capnometry). Respiratory parameters were monitored using sidestream capnometry—the sampling line was connected to the G14 catheter and the end of the cannula was placed in the nasal cavity.

The operated pigs received an antibiotic intramuscularly by literature recommendations [10]. The first injection of amoxicillin (Betamox L.A 150 mg/ml, ScanVet, Poland) at a dose of 15 mg/kg was administered just before the surgery and repeated 48 hours after the surgery. Postoperative analgesic treatment included the administration of meloxicam (0.2 mg / kg, Metacam 5 mg / ml, Boehringer Ingelheim, Germany) for 3 days and once, immediately after surgery, metamizole (50 mg / kg, Pyralgivet 500 mg / ml, Vet-Agro, Poland).

### Operative technique

#### Open surgery

**Fig 1:**
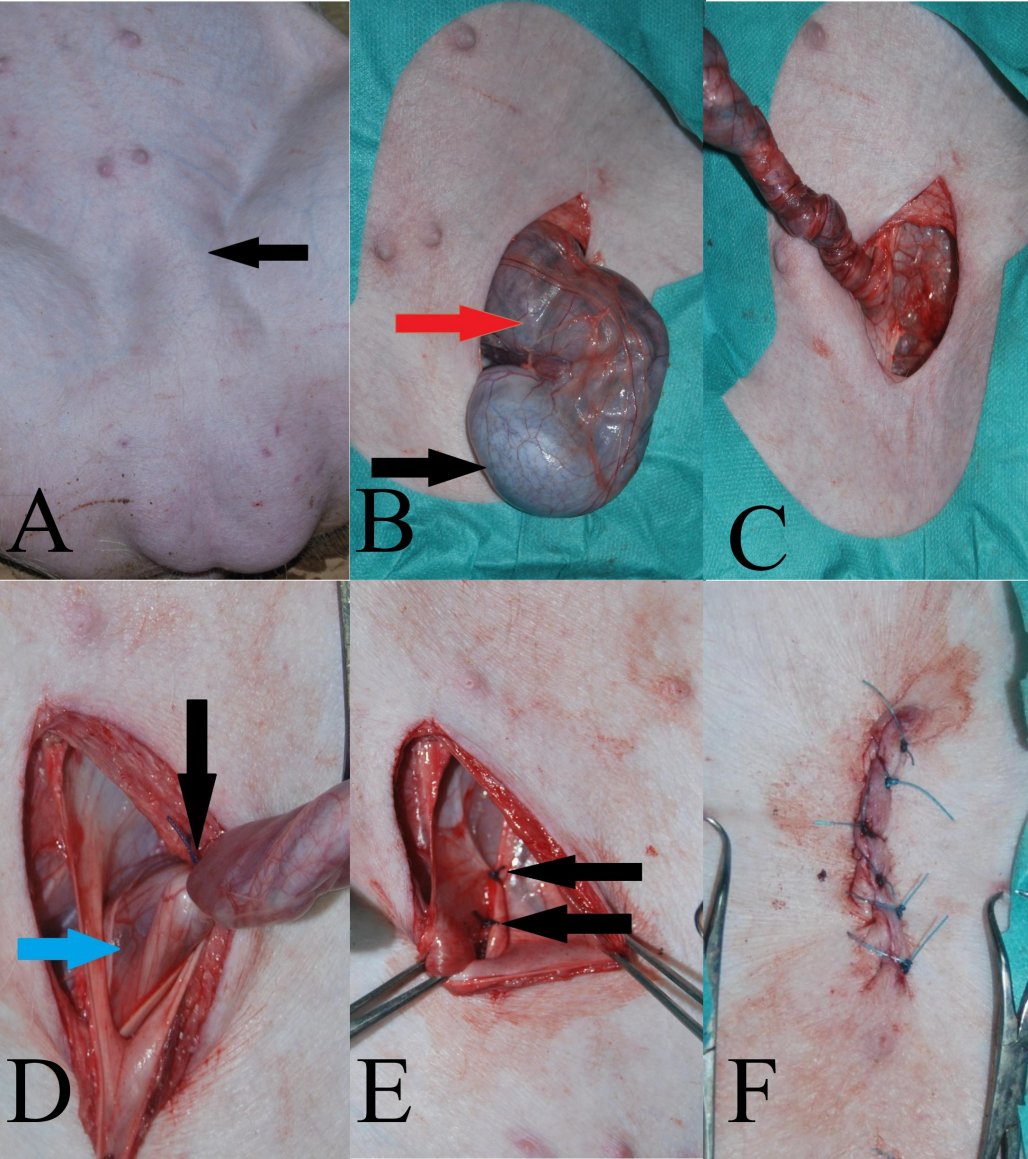
A—Pre-operative appearance of left inguinal hernia—black arrow. B—Intraoperative appearance of the vaginal process with the testicle (black arrow) and hernia contents (red arrow). C—Intraoperative appearance of a twisted vaginal process to drain the contents of the hernia into the abdominal cavity. D—Intraoperative appearance of a twisted vaginal ridge with a ligature just next to the external inguinal canal. E—Intraoperative appearance of the closed external inguinal ring with single broken sutures (arrows). F—The appearance of the postoperative wound at the level of the inguinal canal immediately after the procedure.

Treatment of inguinal hernia in pigs using the technique of open surgery was performed using the method described by Pritchett [8] and Jackson and Cockcroft [24]. For this purpose, the patient was placed in the dorsal recumbency (Fig 1A). After antiseptic preparation of the operating field they were covered with sterile surgical drapes. The procedure began with a skin incision over the inguinal canal with a present hernia. After the skin incision the vaginal process was dissected along with the testicle inside it, the spermatic cord and the abdominal (intestine) organs moved through the inguinal canal. To facilitate the surgical release of these structures during the preparation, manual pressure was applied to the scrotum, moving its contents towards the incision above the inguinal canal (Fig 1B). After dissecting the vaginal process, the testicle inside was grasped and the vaginal process was twisted clockwise until the vaginal process was structured like a twisted rope (Fig 1C). Such action allowed to reduce the contents of the inguinal hernia into the abdominal cavity. The next step in the procedure was to place a ligature on the twisted vaginal process as close as possible to the outer ring of the inguinal canal (polyfilament 0 material, Novosyn, B. Braun, Rubi, Spain) and cut its distal part (Fig 1D). After checking for possible bleeding, the vaginal process stump was pushed inside the abdominal cavity with single interrupted sutures (polyfilament absorbable material 0, Novosyn, B. Braun, Rubi, Spain) over the external ring of the inguinal canal (Fig 1E). The last stage of the procedure was the fixation of the subcutaneous tissue with single interrupted sutures (absorbable poly filament material 0, Novosyn, B. Braun, Rubi, Spain) and the skin with single vertical mattress sutures (non-absorbable monofilament material 0, Dafilon, B. Braun, Rubi, Spain) (Fig 1F). Removal of the second testicle was performed through the scrotal wall, where, after dissection of the vaginal process, a ligature was placed as close as possible to the surgical wound (polyfilament absorbable material 0, Novosyn, B. Braun, Rubi, Spain) and the distal part was cut off. The scrotal wound was closed with single interrupted sutures (monofilament 0 non-absorbable material, Dafilon, B. Braun, Rubi, Spain). The skin sutures from both wounds were removed on the tenth day after the procedure.

#### Percutaneous Internal Ring Suturing (PIRS)

**Fig 2:**
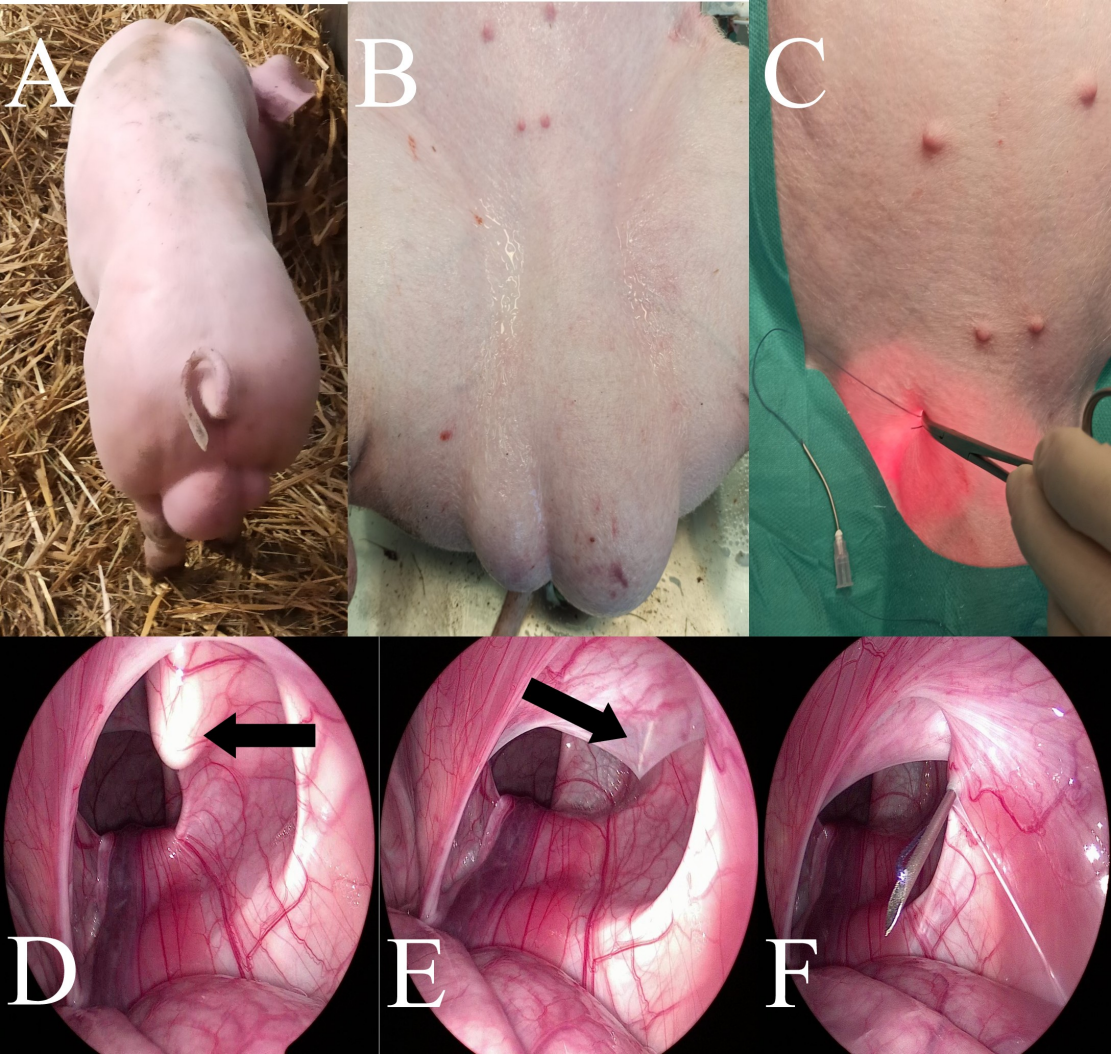
A, B—Preoperative appearance of the left inguinal hernia in the upright position—A and dorsal recumbency—B. C, D—Determination of the place of insertion of the needle and the insertion of the suture over the operated inguinal canal (black arrows). C—external image, B—the laparoscopic image of the abdominal cavity. E—Insertion of the needle into the previously designated place and its passage through the outer half of the inner inguinal canal ring (black arrow)—the laparoscopic image of the abdominal cavity. F—Introduction of the suture through the needle to create a loop—the laparoscopic image of the abdominal cavity.

**Fig 3:**
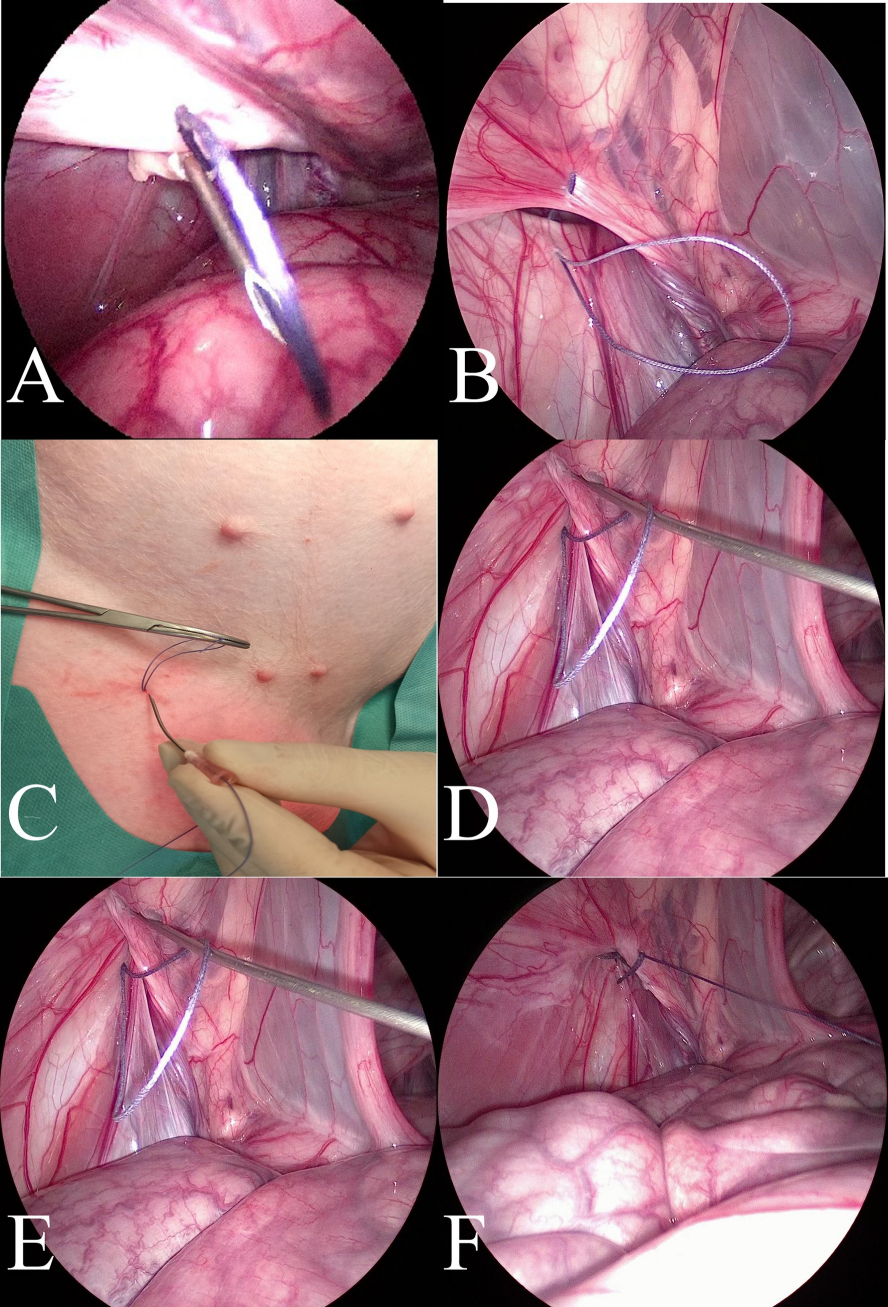
A–Continued introduction of the suture through the needle to create a loop—laparoscopic image of the abdominal cavity. B—View of the loop of the polyester suture after removing the injection needle—the laparoscopic image of the abdominal cavity. C–External image of the needle insertion in the same place where the needle was previously inserted. Before the insertion, the part of the suture at the tip of the needle was cut off from the part of the suture forming the loop in the abdominal cavity. The external portions of the loop were secured with hemostatic forceps. D, E, F—Stages of passing the needle through the medial part of the inner ring of the inguinal canal and introducing the polyester suture placed in the needle through the previously formed loop.

**Fig 4:**
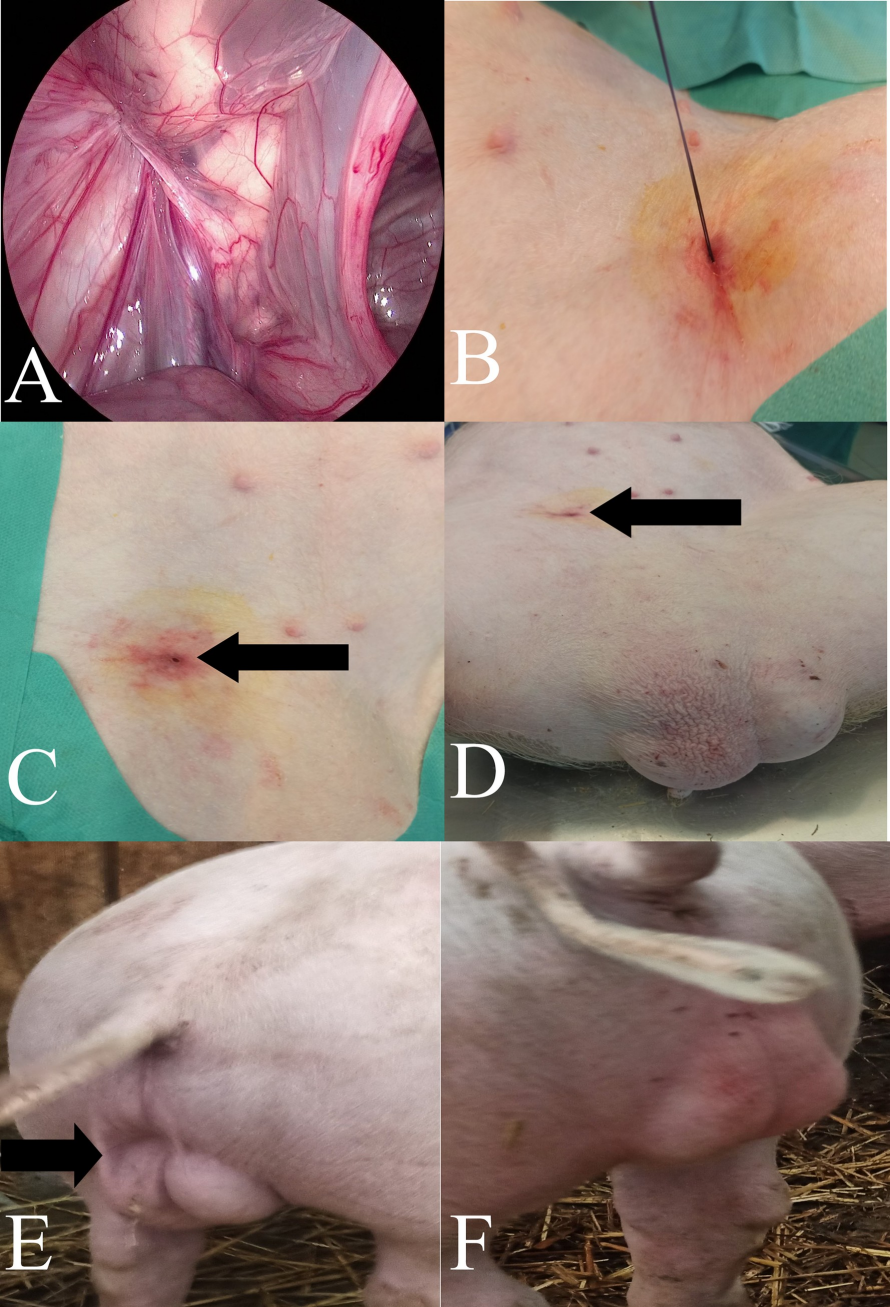
A—Laparoscopic image of the closed inguinal canal. B—Knotting a polyester suture closing the inner ring of the inguinal canal. C, D—Image of postoperative wounds. The black arrow indicates the site of insertion of the needle above the inguinal canal. E, F—the appearance of the scrotum after the PIRS procedure. E—immediately after the procedure, the black arrow indicates excess skin stretched by the contents of the operated inguinal hernia. F—two weeks after the procedure without any excess skin visible before.

Treatments in the PIRS swine group were performed using the original method presented in humans by Patkowski et al. [17] and in dogs by Prządka et al. [18]. All endoscopic equipment used for the laparoscopic procedure with 5mm 30° scope was manufactured by Karl Storz SE & Co. KG (Tuttlingen, Germany). The patient was in dorsal recumbency (Fig 2A and 2B). Pneumoperitoneum (CO2) was established with an open technique by introducing a 5-mm reusable trocar through a longitudinal incision at the umbilicus. Insufflation pressure was between 8–10 mm Hg, based on the patients’ size. The entire peritoneal cavity was inspected. The whole hernia was reduced manually or with the aid of the telescoping tip. All the needle movements were performed from the outside of the body cavity under direct camera control. To choose the location for the needle puncture, the position of the internal inguinal ring was assessed by pressing the inguinal region from the outside with the tip of Pean forceps (Fig 2C and 2D). Under the laparoscopic-guided vision, the 18-gauge injection needle with absorbable 0 polyfilament thread (Novosyn, B. Braun, Rubi, Spain) inside the barrel of the needle was introduced through the abdominal wall entering the abdominal cavity at the middle-upper outline of the internal inguinal ring. With the movements of the tip of the needle, the thread was passed under the peritoneum, over half of the internal ring including a part of the ligament and adjacent tissue (Fig 2E and 2F). The thread was pushed through the barrel of the needle into the abdominal cavity making a loop (Fig 3A). The needle was pulled out, leaving the loop of the thread inside the abdomen (Fig 3B). From outside the pig’s body, one of the thread ends was introduced again into the barrel of the needle and the needle passed through the same skin puncture point to surround the other half of the internal ring with part of the round ligament (Fig 3C). To prevent the vas deferens and testicular vessels from injury, a small space was left above these structures. The end of the thread went through the barrel of the needle into the thread loop, and the needle was withdrawn (Fig 3D and 3E). Next, the thread loop was pulled out of the abdomen with the thread end caught by the loop (Figs 3F and 4A). In this way, the thread was placed around the inguinal ring under the peritoneum and both ends exited the skin through the same puncture point (Fig 4B). The knot was tied to close the internal ring and was placed under the skin (Fig 4C and 4D). The incision in the umbilicus was closed in layers with single absorbable sutures.

### Statistical analysis

Statistical analysis included standard descriptive statistics and normality testing using the Kolmogorov–Smirnov test with the Lilliefors correction. The null hypothesis of normality was not rejected for any of the considered parameters (p> 0.05). To test the differences between the means of the groups, the unpaired t-test was used. The significance level was set to 0.05 in all the above mentioned tests. Only the pigs with unilateral hernia were subjected to statistical analysis because of not sufficient number of subjects in individual groups. Calculations were performed using Statistica 13 (StatSoft, Poland).

## Results

The mean weight was 26.00 ± 10.41 kg in the OS group and 37.40 ± 6.65 kg in the PIRS group, respectively. This difference was statistically significant (p = 0.006). Among the treated pigs, thirteen left-sided, seven right-sided and two bilateral inguinal hernias were found. The diagnosis of inguinal hernias was based on a clinical examination during which deformations of various sizes were found at the level of the inguinal canal, where, through a gentle massage, it was possible to reduce the contents of the hernial sac to the abdominal cavity.

The anaesthesia time was significantly longer in the unilateral PIRS group compared to the unilateral open surgery group (32.40±4.45 vs 26.40±4.93 minutes, respectively, p = 0.01). Whereas, the time of surgery was not significantly different between OS and PIRS group (21.50±4.97 vs 20,90±5.55 minutes, respectively, p>0.05). In one case of bilateral hernia operated with open surgery technique, the time of anaesthesia and surgery was 42 and 35 minutes, respectively. These results were slightly higher than in one bilateral case operated with the PIRS technique (surgery time– 40 minute, anaesthesia time 31 minutes).

All procedures in the PIRS group were possible without the need to convert to open surgery. The animals showed normal motor activity immediately after the end of anesthesia. Among the pigs operated on with the PIRS technique, five of them had medical carbon dioxide in the scrotum of the operated side, with the observed deformation disappearing within a few hours after the surgery (Fig 4E and 4F). At the same time, no other disturbing postoperative complications, such as edema, were observed among the operated pigs, except for one case where clinical symptoms of hernia recurrence were found. It occurred on the sixth day after surgery. Optical trocar postoperative wounds healed by rapid growth leaving a faintly visible linear scar. The site of the needle injection above the inguinal canal was not visible and impossible to indicate during palpation.

In the OS group, all the operated pigs had a scrotal swelling, intensified on the side of the operated inguinal hernia, subsiding after 3–6 days after the procedure. Additionally, there was a slight swelling of the wound edges combined with their delicate redness lasting up to 4 days after the procedure. However, it should be noted that in this group animals were castrated, which can also cause swelling. The skin wounds healed by primary wound healing leaving a linear scar. Hernia recurrence was not reported in any of the operated cases.

## Discussion

An inguinal hernia is the most common congenital defect in pigs, where its left-sided form is dominant [24]. This is also confirmed by the authors of this study, where the left-sided hernia accounted for as much as 65% of all operated unilateral hernias. The lack of surgical treatment of inguinal hernia in pigs leads to serious health consequences for sick pigs and economic losses for their breeders. Evidence of this is as high as 25% mortality rate of fattening pigs affected by the disease and significantly lower daily gains compared to healthy individuals. Also, meat from fattening pigs with an untreated inguinal hernia is classified worse because of post-mortem peritonitis [6, 10].

Reduction or even elimination of surgical castration could be a necessity, which is reflected both in the declarations of European Union Member States as well as in the fulfilment of consumer expectations [25, 26]. The lack of surgical castration when using alternative methods, such as immunocastration, will lead to a situation in which it will be mandatory to surgically treat only individuals with inguinal hernias.

Laparoscopic treatment of inguinal hernia is now a standard in human medicine, gaining more and more popularity in the treatment of companion animals [18]. Pigs as an experimental model for human medicine are often used in the development of new methods of endoscopic surgery, including treatment of inguinal hernia [11–13]. At the same time, based on the knowledge available to the authors, so far none of the methods has been used in pigs as clinical patients. According to the authors, the PIRS technique presented in the paper is the first endoscopic method of treatment of inguinal hernias that can be performed in everyday pigs surgery practice. The presented conclusion results from several facts, the most important of which are the simplicity of performing the described procedure with minimal laparoscopic equipment (a compact set for simple procedures used in farm animals) and a very important issue of economic availability.

Using only one trocar for optics and an 18G injection needle during the entire PIRS procedure significantly reduces the risk of accidental damage to internal organs, which sometimes occurs with the use of additional laparoscopic instruments [18]. Further advantage of the PIRS technique is the straightforward implementation, which, combined with the surgeon’s only basic endoscopic surgery skills, enables its use even in field conditions.

In pigs, during surgical castration, it is recommended to leave the scrotal wound without suturing its edges [10, 24]. In pigs during the OS treatment of unilateral inguinal hernias, it is recommended to carefully examine the second inguinal canal to detect bilateral hernias, where ultrasound examination is a great facilitation [10, 24]. Unfortunately, such additional testing is not always possible under field conditions. Therefore, the authors of this study, after surgical treatment of unilateral inguinal hernia with the open technique (in the OS group), took into account the possibility of undiagnosed small and clinically invisible inguinal hernia on the other side. For this purpose, the second testicle was removed from the cut through the scrotum in the OS group and its wound was sutured. This action was a precaution in the case of possibly bilateral inguinal hernias when one of them was not detected before and during the surgery. Such a procedure is also recommended by other authors, who suggest that in the above-described situations castration should be performed using the closed method [24]. The introduction of the laparoscope optics into the abdominal cavity in the PIRS technique enables the possible diagnosis of an open inguinal canal of the opposite side in cases of only unilateral symptomatic inguinal hernia. On the other hand, the treatment of a bilateral inguinal hernia using the classical surgical technique in pigs requires two separate surgical accesses over each inguinal canal [10]. This is also confirmed by the authors of this study in two cases of bilateral inguinal hernias operated on, with one case for each of the techniques presented in the study. Furthermore, in the PIRS technique through the use of immunocastration and no need for surgical removal of gonads, the risk of eventration is eliminated, which is described as one of the most serious complications of surgical castration resulting from an undiagnosed inguinal hernia [9]. The low invasiveness of the PIRS technique enables to reoperate a possible recurrence of inguinal hernia with the same surgical technique or any other surgical technique known to the surgeon. This is confirmed by the research carried out by the authors, wherein in one case of postoperative recurrence in the PIRS group, open surgery was performed at the owner’s request. In the OS group, a significantly larger difference in the length of postoperative wounds was demonstrated, both in unilateral and bilateral hernias. Complications in the healing of postoperative wounds include swelling and redness of the surgical wound, infection of the operated site, dehiscence of the surgical wound and the aforementioned recurrence of the hernia, leading in extreme cases to eventration. Among the aforementioned complications, the authors found temporary redness and swelling of the postoperative wound and swelling of the scrotum that subsides a few days after the procedure. The current swelling can be reduced by eliminating the dead space during suturing the surgical wound, which, in the case of treating an inguinal hernia with access over the inguinal canal, may be impossible in the area of the scrotum from which the testicle was manually moved to the wound above the inguinal canal [10].

The problem of gonadal circulation disorders as herniorrhaphy serious postoperative complications is raised in both humans and animals [17, 18]. The authors in the PIRS group did not report the above-mentioned complications. Avoid them it is possible by leaving 1–2 mm space above the structures of the spermatic cord while closing the inguinal canal [17, 18]. In children with inguinal hernia treated with PIRS, an accidental puncture of the iliac vessels and temporary bleeding was noted among intraoperative complications [17]. These complications were not reported in the operated pigs, which is similar of treatment of inguinal hernias with the PIRS technique in dogs [18]. It should be noted, however, that both studies on dogs and pigs using the PIRS technique were performed on a relatively small number of animals, which requires further studies on a larger group of animals.

The authors of the study compared the time of anaesthesia and surgery time needed to treat inguinal hernia with the use of open surgery and with the assistance of laparoscopy using the PIRS technique. In pigs with unilateral inguinal hernia, statistically, significant differences were found in terms of weight, time of anaesthesia and wound length. Interestingly, the authors found no statistically significant differences in the time of the surgical procedure itself, despite the previously mentioned differences in the bodyweight of the operated animals. At the same time, the authors would like to point out that they had no final influence on the assignment of operated pigs to particular groups, having to take into account the expectations of the operated animals owners. According to the authors’ knowledge, there is only one experimental work available presenting the time of surgery in the surgical treatment of inguinal hernia with an open technique in pigs [27]. There are many more publications presenting the duration of minimally invasive surgery in the treatment of inguinal hernias in pigs as experimental patients [12, 27–29]. Horgan et al. [27], comparing the strengths and weaknesses of laparoscopic and open mesh inguinal hernia repair, showed that the time needed to repair the inguinal hernia in pigs (12-13kg) with the laparoscopic technique (30–55 minutes) with the use of synthetic mesh was significantly lower compared to the open technique (45–75 minutes). A study on larger (70 kg) pigs, comparing two methods of fixing hernial meshes, showed that the operative time was significantly lower in the self-adhesive group: 23 minutes (15–32) versus fixed in place with staples group: 31 minutes (21–40) [28]. A similar treatment time of 15–20 minutes was obtained by Schulze et al. [29] performing a laparoscopic closure of the inguinal canals and fixing the hernia meshes with a tissue adhesive. The literature also describes the possibility of treating inguinal hernia with the NOTES technique in pigs (35–40 kg) where the time needed to complete the entire procedure was between 65 and 120 minutes. The mean time of procedures in the presented study, performed using the open surgery technique, was 20.90 ± 5.55 minutes, while the procedures performed using the PIRS technique were 21.50 ± 4.97 minutes, with the mean weight of pigs in the OS of 26.0 kg group and 37.40 kg for the PIRS group. The presented mean duration of an open surgery procedure in this study is almost three times shorter than that presented by Horgan et al. [27] When comparing the authors’ meantime using the PIRS technique in pigs, it should be noted that it is similar or lower compared to the other available data presented above, apart from the operated pigs’ weight (12-70kg). At the same time, the results of the meantime of PIRS treatment in pigs obtained by the authors are comparable to the results obtained in dogs using the same surgical technique [18]. Comparing the duration of PIRS and open surgery in the treatment of bilateral inguinal hernias, the authors only had one case in each group. However, analysing the obtained results, it can be concluded that both the time of anaesthesia and the time of the procedure were similar in both groups.

Another issue is the type of suture material used in the work. Most of the available studies describing the use of the PIRS technique in the treatment of inguinal hernias recommend the use of non-absorbable material [17, 18]. The authors of this study used absorbable material with a very good clinical effect. The choice of material was dictated by practical considerations resulting from the necessity of its absorption by the organisms of the operated pigs before reaching the slaughter weight. This is also confirmed in the literature, where an absorbable material is recommended in pigs with an open herniorrhaphy technique [8, 10, 24].

The authors of the study, comparing the two presented surgical techniques, showed a significantly shorter length of the postoperative wound in the PIRS group, amounting to only half a centimetre. A smaller surgical wound reduces the possibility of postoperative complications, such as infection or dehiscence, which may be important in farm conditions. Of course, it should be critically emphasized that in the PIRS technique, access to the abdominal cavity is always performed, which is not required in the open technique. However, open-technique inguinal hernia procedures sometimes end with a complication related to the intraoperative accidental opening of the vaginal process, and thus the opening of the abdominal cavity. According to the authors’ experience, such a situation occurs much more often in very young individuals. Although PIRS treatments were performed on pigs weighing an average of 37 kg, the authors are convinced that this procedure is possible in much lighter pigs. This is confirmed by studies carried out on dogs where the smallest operated dog weighed 2.1 kg [18]. Nevertheless, according to the authors, the study requires not only the technical possibility of performing the PIRS procedure in small piglets during the recommended castration date [10] but also the possibility of their safe anaesthesia for laparoscopic procedures in farm conditions.

The last issue discussed in this paper is postoperative recurrence. The authors of the tested animals recorded only one recurrence in the PIRS group, which was stocked classically at the owner’s request. At the same time, according to the authors’ knowledge, no precise data are presenting the frequency of postoperative recurrences in pigs using the open technique. According to the literature on human medicine it can be assumed that the percentage of postoperative relapses will be greater than that obtained in the authors’ results. According to the authors, such a result is related to a relatively small number of animals tested. The frequency of recurrences after inguinal hernia surgery with the laparoscopic technique in children is slightly higher than with the open technique. At the same time no postoperative recurrences were shown in children under 3 months of age. The lack of recurrences in the youngest children is probably because of the body’s natural biological ability to close the inguinal canal after its narrowing with the PIRS technique [17].

## Conclusions

The results of the conducted research show that laparoscopic-assisted herniorrhaphy using the PIRS technique is an effective treatment method of inguinal hernia in pigs. The effectiveness of the PIRS technique is comparable to the classical method of open surgery currently used in pigs. According to the authors, the PIRS technique presented for the first time in pigs may be an alternative in the treatment of inguinal hernias in males not subjected to surgical castration. At the same time, according to the authors, further studies are needed on a larger group of pigs with inguinal hernia, including small piglets, females and pet pigs whose life span is longer, enabling long-term clinical evaluation.
